# Supplementary material for: Determinants of Undernutrition and Associated Factors of Low Muscle Mass and High Fat Mass among Older Men and Women in the Colombo District of Sri Lanka
Source: Geriatrics (Basel). 2022 Feb 28;7(2):26. doi: 10.3390/geriatrics7020026 (PMC8938783; doi:10.3390/geriatrics7020026)
Supplement: Supplementary file 1 [file geriatrics-07-00026-s001.zip › geriatrics-1586884-Supplementary Material S1.pdf]

Annex II

Confidential

Undernutrition, dietary pattern, associated factors and effectiveness of a nutrition education intervention in improving undernutrition among elderly in Colombo District

Questionnaire

DS Division: Serial no.

GN Division:

Name of the elderly:

Address: Date:

Part one - Assessment of factors associated with Undernutrition

Part A- Socio-Demographic factors

1. Date of birth Day……… Month……. year…………

Age in years ………………………

2. Sex

1. Male	
2.Female	

3. Ethnicity

1.Sinhala	
2.Tamil	
3.Muslim	
4.Burger	
5.Other	

4. Religion

1.Buddhist	
2.Christian	
3.Hindu	
4.Islam	
5.other	

5. Marital status

1.Married	
2.Widowed	
3.Divorced	
4.Unmarried	
5.Separated	
6.Living together	

6. What is your living environment?

1.Urban - Residential	
2.Urban – Slum	
3.Rural	
4.Estate	

7. What is your highest educational achievement?

1.No schooling	
2.Grade 1-5	
3.Grade 6-10	
4.G.C.E O/L passed	
5. G.C.E. A/L passed	
6.Degree and Postgraduate	

8. What is your employment status?

1.Currently employed	
2.Retired	
3.Never employed	

9. What is your monthly income in rupees?

1.No income	
2.< 5000	
3.5000 – 10000	
4.10,001 – 15, 000	
5.>15, 000	

10. What is your Source of income? (can mark more than one)

1.Employment / pension	
2.Assets / Bank accounts	
3.House rentals	
3.Family members support	
4.Relatives support	
5.Government assistance	

11. How many others living with you & the relationship with you? Total…….

Relationship
1.
2.
3.
4.
5.
6.
7.
8.
9.
10.

12. Who is your primary care giver?

1.husband/ wife	
2.Son/Daughter	
3.Son in law/Daughter in law	
4.Siblings	
5.Grand children	
6.Relative	
7. Other	

13. Do you look after your grandchildren?

1.Yes	
2.No	

14. What is your level of satisfaction on family support?

1.Very satisfied	
2.Satisfied	
3.Dissatisfied	

Part B- Factors associated with Health status and Disabilities

15. Are you suffering from following chronic medical conditions?

1.Diabetes Mellitus	
2.Hypertension	
3.Heart diseases	
4.Renal failure	
5.Asthma/COPD	
6.Convulsions	
7.Any other	

16. Were you hospitalized during the past 3 months?

1.Yes	
2.No	

17. If yes, duration of hospitalization……………….

18. Where do you go to seek treatment? (Mark the most frequently visited place)

1.Government – western Institution	
2.Government – indigenous Institution	
3.Private – western institution	
4.Private – indigenous institution	
5.Spiritual/ religious activities	
6.Other	

19. How many drugs do you use for co- morbidities per day?.................

20. Are you having food allergies?

1.Yes	
2.No	

21. Are you having disabilities?

1.Yes	
2.No	

22. What are the physical disabilities you are having and mark whether you are using disability aids?

Physical disability	Present or not
1.Hearing	
2.Smell	
3.Taste	
4.vision	
5.Chewing	
6.Musculoskeletal problems	
7.Any other	

23. What are the Psychological disabilities you are having and mark whether you are taking treatment?

Psychological disability	Present or not
1.Depression	
2.Schizophinia	
3.Any other	

Part C – Factors associated with behaviors

24. Do you currently smoke?

1.Yes	
2.No	

25. Are you currently using alcohol?

1.Yes	
2.No	

26. If yes, what is the type and amount (per week) of alcohol you are consuming?

Type of alcohol		Amount
1.Arrack		
2.Beer		
3.Kasippu		
4.Other		

27. Are you chewing betel?

Yes	
No	

28. If yes, what is the frequency per day?.................

Part Two – Assessment of factors associated with dietary pattern

29. How much of responsibility do you have for the following?

	Little or none	Average	Most or all
1.Food shopping			
2.Planning meals			
3.Preparing meals			

30. In a typical week, where are your meals prepared mostly?

	At home	Out	Do not eat meals
1.Breakfast			
2.Lunch			
3.Dinner			

31. Do you skip meals?

1.Yes	
2.no	

32. How do you receive nutritional advises? (Can give more than one response)

1.Not receiving	
2.General practitioner	
3.Hospital	
4.Public Health Midwife	
5.Others specify….	

33. Availability of a home garden?

1.Yes	
2.No	

Part Three – Assessment of the dietary pattern

24 hour dietary recall sheet

MEAL	Items consumed	Amount
**Break fast**		
**Morning snack**		
**Lunch**		
**Afternoon snack**		

**Dinner**		
**Other**		

Part Four- Assessment of nutritional status

Anthropometric measurements and Body composition measurements

	First time	second time	Average
**Weight**			
**Height**			
**Arm span length**			
**Mid upper arm circumference**			
**Body fat percentage**			
**Body muscle percentage**			
