# Supplementary material for: Determinants of Undernutrition and Associated Factors of Low Muscle Mass and High Fat Mass among Older Men and Women in the Colombo District of Sri Lanka
Source: Geriatrics (Basel). 2022 Feb 28;7(2):26. doi: 10.3390/geriatrics7020026 (PMC8938783; doi:10.3390/geriatrics7020026)
Supplement: Supplementary file 1 [file geriatrics-07-00026-s001.zip › geriatrics-1586884-Supplementary Material S2.pdf]

Supplementary material 3

Operationalized definition of variables

Variable	Operational definition	Type of the variable
**Age**	Number of years completed for the last birth day. Verified by the birth certificate/ National ID. Later categorized into a binary variable as less than 70 years and >=70 years.	Continuous
**Sex**	Male or Female	Binary
**Ethnic Group**	Recorded as Sinhala, Tamil, Muslim, Burger and other. Later categorized in to a binary variable as Sinhala and others.	Categorical
**Marital Status**	Recorded as Married – where the legally married older person is living with the spouse, Widowed – when the spouse of the older person is dead, Divorced when separated legally, separated when the couple is permanently separated but not legally and unmarried as never married older person. Later categorized into a binary variable as married and divorced/widowed/unmarried/separated	Categorical
**Living Environment**	The place where a person is resident at the time of survey and recorded as urban - residential, Urban-Slum, rural or estate according to the GN division breakdown by the Department of Census and Statistics. Later categorized into a binary variable as urban and others.	Categorical

**Highest Educational achievement**	Assessed by considering the highest education level achieved by the older person.<br>No schooling<br>Grade 1 – 5<br>Grade 6 – 10<br>G.C.E O/L passed<br>G.C.E A/L passed<br>Degree/postgraduate<br>Later categorized into a binary variable as no education/up to grade 5 and above grade 5	Categorical
**Employment status**	Employment status was recorded as Currently employed when the older person is economically active during the survey and Retired, when economically active person but currently unemployed and Never employed. Later categorized into a binary variable as currently unemployed and not employed.	Categorical
**Monthly income**	Total monthly income of the older person was considered and assessed according to the perception of the respondent as having a monthly income and not having a monthly income.	Categorical
**Chronic illness/condition**	Common morbid conditions (DM/HT/HD/COPD) among the older people which were diagnosed by a physician & diseases written in the patient's record/self-reported was considered as having a chronic illness.	Categorical
**Disabilities**	Presence of the disabilities of vision/hearing/ chewing/musculoskeletal and whether taking treatment, reported by the participant, care giver or by diagnosis cards/ clinic records. Musculoskeletal	Categorical

	conditions were defined as “Injuries/disorders of muscle/bone with limitation of movements.”	
**Betel chewing**	Current usage as reported by the participant, as “yes” and “no”.	Categorical
**Responsibility on food shopping, planning and preparing meals**	Reported according to the perception of participants and was recorded as little or none, average and most as they perceive. Later categorized into a binary variable as little/no responsibility and average/most responsibility with food shopping, planning meals and preparing meals.	Categorical
**Skipping meals**	If the participant skipped a meal even once during the past 2 weeks, he was recorded as “yes”.	Categorical
**Method of nutritional advises**	Was recorded as not receiving, Receiving from GP, Hospital, PHM or by mass media as the main method. According to the response by the respondent.	Categorical
