# Supplementary material for: Determinants of Undernutrition and Associated Factors of Low Muscle Mass and High Fat Mass among Older Men and Women in the Colombo District of Sri Lanka
Source: Geriatrics (Basel). 2022 Feb 28;7(2):26. doi: 10.3390/geriatrics7020026 (PMC8938783; doi:10.3390/geriatrics7020026)
Supplement: Supplementary file 1 [file geriatrics-07-00026-s001.zip › Supplementary Tables.pdf]

Supplementary Tables

Supplementary Table S1: Diagnosis criteria of undernutrition

ASPEN Criteria	ESPEN Criteria	MNA Criteria
Low energy intake	BMI<18.5Kg/m^2 with weight loss	*Reduced food intake
Weight loss	BMI<18.5Kg/m^2 with reduced fat-free mass	*Weight loss
Loss of muscle mass		*Presence of Acute disease
Loss of subcutaneous fat		*Mobility
Fluid accumulation		BMI (< 18.5 kg/m^2)
Hand grip strength		*Neuropsychological problems

* Presence of symptoms during past 3 months

Supplementary Table S2: Previous studies showing the different tools used and the key findings

Country/District	Study Ref	Sample size	Prevalence of undernutrition	Year	Tool
Sri Lanka Colombo	[11]	452	20%	2012	Anthropometry
Sri Lanka Galle	[30]	396	0.5%,<br>at risk -30.8%	2017	MNA
Sri Lanka Kandy	[12]	999	12.7%	2017	MNA
Sri Lanka Mathale	[13]	3194	38.4%	2002	Anthropometry
Sri Lanka Mannar	[14]	200	45%	2007	Anthropometry
India	[26]	190	19.47 %	2016	MNA
Nepal	[27]	242	24%	2017	MNA
France	[28]	692	Urban-7.4%<br>Rural-18.5%	2014	MNA
Turkey	[29]	1030	16%	2015	MNA

Supplementary Table S3: Criteria used to describe an older person as undernourished (overall)

**Criteria**	**Cut-off value for undernutrition in men**	**Cut-off value for undernutrition in women**
BMI	< 18.5 Kg.M^2	< 18.5 Kg.M^2
MUAC	≤ 23.0 cm	≤ 22.0 cm
Body fat percentage	< 14.0 %	< 26.0%
Body muscle percentage	< 34.4%	< 26.6%

Supplementary Table S4: Comparison of key findings of the present study and previous studies

**Key finding**	**Present study**	**Previous studies**
**Prevalence of undernutrition**		
	35.3%	20%-Colombo-Sri Lanka [11]<br>38.4% - Mathale-Sri Lanka [13]<br>19.5% in India [26]<br>24.0% in Nepal [27]<br>7.5% in France [28]<br>16% in Turkey [29]
**Factors associated with undernutrition**		
	Age ≥70 years -associated with low muscle mass and high fat mass	**Age ≥60 years**<br>Galle-Sri Lanka [30]<br>Kandy district-Sri Lanka [12]<br>**Age ≥70 years**

		Northwest Ethiopia [35]<br>France [28]
	Presence of Diabetes Mellitus associated with low muscle mass and high fat mass	Associated with undernutrition [11]
	Disability in chewing with high fat mass in women	Associated with tooth loss [36]

Supplementary Table S5: Distribution of older men and women according to 4 categories of muscle mass and fat mass

	**% (95% CI) among older Women**<br>**555/800**	**% (95% CI) among older Men**<br>**245/800**
High fat mass and low muscle mass	4.3% (2.6-5.7)<br>N=24	15.1% (10.6-19.5)<br>N=37
Low fat mass and low muscle mass	21.4% (17.7-24.8)<br>N=119	10.6% (6.9-14.6)<br>N=26
High fat mass and high muscle mass	70.6% (66.8-74.3)<br>N=392	11.8% (7.7-15.8)<br>N=29
Low fat mass and high muscle mass	3.6% (2.0-5.1)<br>N=20	62.4% (56.3-68.4)<br>N=153
